# Diurnal Variations and Test–Retest Reliability of Resting‐State Functional MRI Metrics

**DOI:** 10.1002/hbm.70590

**Published:** 2026-07-07

**Authors:** Bowen Guo, Kaikai Yan, Zhihui Liu, Yao Deng, Shanna Fu, Weiwei Zhao, Jing Xu, Caihong Jiang, Ruiwen Tao, Xing Chen, Mengmeng Wang, Tianxin Mao, Hengyi Rao

**Affiliations:** ^1^ Department of Psychology, School of Humanities and Social Sciences University of Science and Technology of China Hefei China; ^2^ Center for Magnetic Resonance Imaging Research & Key Laboratory of Brain‐Machine Intelligence for Information Behavior (Ministry of Education and Shanghai), School of Business and Management Shanghai International Studies University Shanghai China; ^3^ State Key Laboratory of Cognitive Neuroscience and Learning & IDG/McGovern Institute for Brain Research Beijing Normal University Beijing China; ^4^ Department of Psychology Zhejiang Sci‐Tech University Hangzhou China; ^5^ Business School, NingboTech University Ningbo China; ^6^ Center for Functional Neuroimaging, Department of Neurology University of Pennsylvania Philadelphia Pennsylvania USA; ^7^ Unit for Experimental Psychiatry, Division of Sleep and Chronobiology, Department of Psychiatry University of Pennsylvania Philadelphia Pennsylvania USA

**Keywords:** diurnal variation, intraclass correlation coefficient (ICC), resting‐state fMRI, test–retest reliability, time‐of‐day

## Abstract

Resting‐state fMRI (rs‐fMRI) is widely used to assess intrinsic brain activity, yet concerns about its test–retest reliability and reproducibility persist. Circadian rhythms strongly influence brain physiology, but their impact on rs‐fMRI reliability remains poorly understood. In this study, we scanned 39 healthy young adults six times within a single day (08:00–20:00) under standardized conditions. For each session, we computed four common rs‐fMRI metrics, including amplitude of low‐frequency fluctuations (ALFF), wavelet‐transformed ALFF (wALFF), fractional ALFF (fALFF), and regional homogeneity (ReHo), and assessed reliability using intraclass correlation coefficients (ICCs). ReHo showed relatively higher and more stable reliability across sessions, whereas amplitude‐based metrics, particularly fALFF, exhibited greater diurnal variation. Both network‐level and region‐specific analyses revealed low reliability in the limbic and subcortical structures, with a mid‐morning dip at 10:00. Moreover, ICCs for ALFF, wALFF, and fALFF declined with increasing inter‐scan intervals, whereas ReHo remained robust. These findings demonstrate diurnal fluctuations in rs‐fMRI reliability, with different metrics exhibiting distinct temporal stability profiles. We recommend that scan timing and circadian influences should be explicitly considered in the design, analysis, and interpretation of future rs‐fMRI studies.

## Graphical abstract

Regional homogeneity (ReHo) exhibited the highest within‐day reliability, whereas fractional amplitude of low‐frequency fluctuations (fALFF) showed the lowest. The limbic network demonstrated the lowest reliability among the networks, with a notable mid‐morning dip at 10:00.

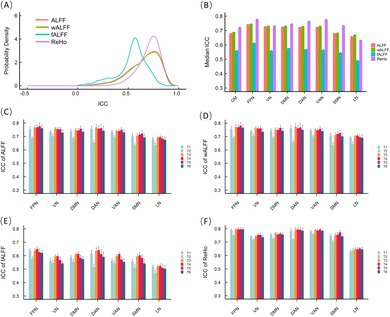

## 1 Introduction

Magnetic resonance imaging (MRI) has greatly advanced our understanding of the human brain, enabling precise mapping of both structure and function (Marek et al. 2022; Raichle et al. 2001). Functional MRI (fMRI), particularly resting‐state paradigms, has become a cornerstone of cognitive and clinical neuroscience (Buckner et al. 1996; Wagner et al. 1998). The validity of fMRI inferences depends on reliable and reproducible measures (Ekhtiari et al. 2024; Marek et al. 2022; Mekbib et al. 2024; Schwartz et al. 2019), yet reproducibility concerns persist, with false‐positive rates reported as high as 10%–40% (Carp 2013; Wager et al. 2009). Test–retest studies have identified multiple sources of variability, including scanner hardware, acquisition protocols, analysis pipelines, and subject‐level factors such as motion (Andellini et al. 2015; Elliott et al. 2020, 2021; Haller et al. 2018; Herting et al. 2018; Marek et al. 2022; Yan et al. 2013). However, some potentially critical sources of intra‐individual variability remain under‐explored. In particular, the time of day when scans are acquired may systematically influence brain activity but has not been fully characterized in the context of fMRI reliability (Guo et al. 2024).

Time‐of‐day is known to exert broad effects on physiology, neural, and cognitive functions (Deota et al. 2025; Goel et al. 2013; Guo et al. 2024; Hartsock et al. 2024; Klerman 2005; Lange et al. 2010; Mohd Azmi et al. 2021; Shannon et al. 2013; Serin and Acar Tek 2019; Truong et al. 2023). According to the two‐process model of sleep–wake regulation, circadian rhythms and homeostatic sleep drive jointly regulate arousal and brain activity (Borbély 2002, 2022). Empirical evidence shows robust diurnal modulations of brain function. For example, resting‐state blood‐oxygen‐level‐dependent (BOLD) fluctuations vary across the day (Orban et al. 2020; Vaisvilaite et al. 2022; Vaisvilaite et al. 2023), with reductions in local activity observed during nocturnal periods (Xing et al. 2023). Cerebral blood flow (CBF) and neuroendocrine signals, including cortisol, also follow circadian cycles, with perfusion in regions such as the anterior cingulate tracking hormonal rhythms (Braun et al. 1997; Elvsåshagen et al. 2019; Hodkinson et al. 2014). Task‐based studies further demonstrate circadian influences on auditory working memory (Vandewalle et al. 2011), cortical responsiveness to sleep pressure (Muto et al. 2016), and reward‐related striatal circuits (Byrne et al. 2017). Collectively, these findings suggest that scan timing could systematically affect rs‐fMRI outcomes, yet its impact on the test–retest reliability of resting‐state measures remains poorly understood.

Among common rs‐fMRI metrics, ALFF and its derivatives quantify the power of spontaneous BOLD oscillations, whereas ReHo measures local voxel‐to‐voxel synchrony (Zang et al. 2004; Zou et al. 2008). Variants such as wALFF, which employs continuous wavelet transformation to improve time‐frequency resolution (Luo et al. 2020; Sifuzzaman et al. 2009; Torrence and Compo 1998), and fALFF, which normalizes ALFF to the full frequency spectrum (Liang et al. 2021), are designed to enhance noise robustness and sensitivity to transient signals. These complementary measures are widely applied in clinical, cognitive, and interventional research (e.g., Lv et al. 2018; Yang et al. 2007). Prior studies have reported moderate‐to‐high test–retest reliability for ALFF and ReHo across days to weeks, with ICC often exceeding 0.4 even across long intervals (Zuo, Di Martino, Kelly, et al. 2010; Zuo, Kelly, Adelstein, et al. 2010; Zuo, Kelly, Di Martino, et al. 2010; Zuo et al. 2012, 2013). Reliability may vary by region, with high stability in the posterior cingulate and low in the hippocampus (Park et al. 2012). Our recent work demonstrated excellent within‐day reliability of absolute CBF measured via arterial spin labeling (ASL) across six sessions both at resting state and during task performance (Guo et al. 2024). However, the intra‐day reliability of ALFF, wALFF, fALFF, and ReHo, as well as their sensitivity to scan timing, remain unclear.

The present study addresses this gap by systematically examining the within‐day test–retest reliability of rs‐fMRI metrics. Forty healthy adults were scanned at six time points under standardized conditions. We computed whole‐brain, network‐level, and region‐level ALFF, wALFF, fALFF, and ReHo for each session and quantified reliability using ICCs. Specifically, we examined (1) overall reliability and spatial distributions of each metric, (2) how reliability varies with scan timing, and (3) the influence of inter‐scan interval. To our knowledge, this is the first within‐day design to jointly evaluate multiple ALFF variants and ReHo. Our findings reveal pronounced diurnal effects on rs‐fMRI reliability and identify ReHo as the most robust measure, with reliability notably lowest around 10:00. These results highlight the importance of scan timing in experimental design and support ReHo as a particularly stable metric for studies spanning different times of day.

## 2 Materials and Method

### 2.1 Participants

Forty healthy, right‐handed adults (24 females; mean age = 21.68 ± 2.46 years, range = 18–27) were initially recruited for this study via campus advertisements and online postings. All participants met the following inclusion criteria: (1) habitual sleep duration of 7–9 h per night and stable sleep–wake schedules (bedtime between 22:00 and 24:00 and wake time between 06:00 and 08:00), confirmed by sleep diaries and wrist‐worn actigraphy during the 7 days preceding the study; (2) no history of psychiatric or neurological disorders (e.g., anxiety, depression, insomnia); (3) no recent engagement in circadian‐disruptive behaviors (e.g., night shift work, transmeridian travel, or substance use); (4) normative chronotype and body mass index (BMI); (5) abstinence from caffeine and alcohol for at least 7 days prior to participation; and (6) no contraindications for MRI or self‐reported sensory impairments.

Screening procedures involved validated questionnaires: Pittsburgh Sleep Quality Index (PSQI; Buysse et al. 1989), Insomnia Severity Index (ISI; Morin et al. 2011), Morningness‐Eveningness Questionnaire (MEQ; Horne and Ostberg 1976), Self‐Rating Anxiety Scale (SAS; Zung 1971), Self‐Rating Depression Scale (SDS; Zung 1965), and Beck Depression Inventory (BDI; Beck and Beamesderfer 1974). Participants with abnormal scores or noncompliance with sleep requirements were excluded. One participant was excluded from subsequent analyses due to excessive head motion, resulting in a final sample of 39 participants. The study was approved by the Ethics Committee of Shanghai International Studies University and adhered to the Declaration of Helsinki (Approval No.: 2019 BC000). Written informed consent was obtained from all participants prior to study enrollment.

### 2.2 Experimental Design

Each participant underwent six rs‐fMRI sessions across a single day, scheduled at 08:00, 10:00, 13:00, 15:00, 18:00, and 20:00. Each session included an 8‐min resting‐state BOLD scan, during which participants fixated on a central cross with eyes open. Before each scan, participants were explicitly instructed to keep their eyes open and remain awake throughout the scanning session, and they were monitored using an eye‐tracking system to ensure that they stayed awake during the entire scan (Zou et al. 2015). High‐resolution structural scans were also acquired for co‐registration and spatial normalization.

To minimize confounding factors, participants followed a standardized protocol on the scanning day, including scheduled meals, abstention from caffeine and physical exertion, and engagement only in sedentary activities (e.g., reading or watching television) between scans. Scanning was performed at the MRI Research Center of Shanghai International Studies University under continuous supervision. Related findings from this broader project have been published elsewhere (Guo et al. 2024).

### 2.3 Imaging Data Acquisition and rs‐fMRI Metric Calculation

All MRI scans were conducted using a 3 T Siemens MAGNETOM Prisma scanner (Siemens Medical Systems, Erlangen, Germany) equipped with a 64‐channel head coil. Resting‐state BOLD images were acquired using a gradient‐echo EPI sequence (TR = 2000 ms; TE = 30 ms; flip angle = 90°; FOV = 192 × 192 mm^2^; slice thickness = 3 mm; 33 interleaved slices without gaps; 240 volumes per session). High‐resolution T1‐weighted images were obtained with a 3D MPRAGE sequence (TR = 2530 ms, TE = 2.96 ms, TI = 1100 ms, FOV = 192 × 192 mm^2^, matrix size = 256 × 256, voxel size = 0.9 × 0.9 × 0.9 mm^3^, 192 slices).

Pre‐processing was performed using DPABISurf v2.1 (Yan et al. 2021), which incorporates the fMRIPrep pipeline (Esteban et al. 2019). Key preprocessing steps included: (1) Initial data preparation: The first 10 time points were removed to allow for signal stabilization. All data were converted to BIDS format (Gorgolewski and Poldrack 2016) and processed using fMRIPrep 1.5.0. (2) Anatomical data pre‐processing: T1‐weighted (T1w) images were corrected for intensity non‐uniformity using N4BiasFieldCorrection (Tustison et al. 2010) and served as the T1w reference. Skull stripping was performed using the antsBrainExtraction workflow (Avants et al. 2008), with the OASIS30ANTs template. Tissue segmentation into cerebrospinal fluid (CSF), white matter (WM), and gray matter (GM) was performed using FAST (FSL 5.0.9) (Zhang et al. 2001). Spatial normalization to MNI152NLin2009cAsym template was achieved via nonlinear registration with antsRegistration (ANTs 2.3.3). (3) Functional data pre‐processing: A reference BOLD volume and its skull‐stripped version were generated using fMRIPrep. The BOLD reference was co‐registered to the T1w reference using boundary‐based registration (bbregister, FreeSurfer) (Greve and Fischl 2009). Slice timing correction and motion correction were applied (Cox and Hyde 1997; Power et al. 2014), followed by resampling to a 2 × 2 × 2 mm^3^ voxel size. The BOLD time series was then transformed into MNI152NLin2009cAsym space. (4) Nuisance regression and motion control: Nuisance signals from 24 motion parameters (Friston et al. 1996), WM and CSF were regressed out. Participants with excessive head motion (translation > 2.5 mm, rotation > 2.5°, or mean frame‐wise displacement > 0.3 mm) were excluded. Linear trends were removed to correct for signal drifts.

rs‐fMRI metrics were computed using DPABI v7.2 (Yan et al. 2016) and RESTplus v1.25 (Jia et al. 2019). ALFF was defined as the sum of amplitudes within the 0.01–0.10 Hz band. wALFF was calculated using continuous wavelet transformation, summing the coefficients across time points and averaging within the 0.01–0.10 Hz band (Luo et al. 2020; Sifuzzaman et al. 2009; Torrence and Compo 1998). fALFF was the ratio of ALFF to the total signal amplitude across all detectable frequencies (Liang et al. 2021). ReHo was computed using the Kendall's coefficient of concordance (KCC) for each voxel and its neighboring voxels after band‐pass filtering (0.01–0.10 Hz) (Liu et al. 2019; Zuidema et al. 2024). All metric maps were standardized by dividing each voxel's value by the global mean. Spatial smoothing with a full‐width at half‐maximum (FWHM) of 6 mm was applied to enhance the signal‐to‐noise ratio (Zuidema et al. 2024). It should be noted that although Prescan procedures, detrending, and filtering were used to stabilize the signal (Greve et al. 2011; Wang et al. 2014), the lack of concurrent hardware QA data on the scanning day is an inherent limitation of this study.

### 2.4 Test–Retest Reliability Analysis Across the Day

Test–retest reliability of rs‐fMRI metrics was quantified using the ICC based on McGraw and Wong's (1996) framework, which incorporates between‐subject (BMS), within‐subject (JMS), and residual error (EMS) components. Analyses were performed across the six repeated sessions (*k* = 6) from 39 participants in the final sample (*n* = 39). We utilized the ICC(1) formulation (one‐way random effects model) to assess the reliability of brain activity (McGraw and Wong 1996). Before ICC estimation, voxel values were adjusted for covariates by regressing out age (z‐scored) and sex within each session. ICC was then computed on the resulting residuals. Reliability was interpreted using the following thresholds: poor (0.00–0.39), fair (0.40–0.59), good (0.60–0.74), and excellent (0.75–1.00) (Hodkinson et al. 2013). Voxel‐wise ICC maps and temporal ANOVA were computed using RESTplus v1.25 and custom MATLAB 2021a scripts.

Voxel‐wise ICC values were constructed into histograms to provide a qualitative comparison of images. The differences in ICC distributions between metrics were statistically assessed through the Friedman test and post hoc Wilcoxon signed‐rank tests (Yan et al. 2013). Median ICC within each region was selected as the ROI‐level summary metric, given its robustness to smoothing and parcellation granularity (Caceres et al. 2009). We first calculated ICCs across the whole‐brain GM, using tissue probability masks from DPABI with a GM threshold > 20%. To further evaluate reliability across canonical functional systems, we extracted ICCs from Yeo's et al. (2011) seven‐network atlas: visual (VN), somatomotor (SMN), dorsal attention (DAN), ventral attention (VAN), frontoparietal (FPN), limbic (LN), and default mode networks (DMN). It should be noted that ReHo (reflecting spatial synchronization) and ALFF‐derived metrics (reflecting oscillation amplitude) assess distinct physiological properties of the BOLD signal. Therefore, our cross‐metric comparisons focus primarily on the trends of diurnal variation and temporal stability, rather than on direct comparisons of absolute ICC scores.

### 2.5 Time Point and Interval‐Based Analyses

To assess the reliability of each time point, ICCs were calculated between each pair of the six scanning sessions, yielding 15 ICC comparisons per metric. For each time point, an average ICC was calculated by averaging the five pairwise ICCs involving that session (e.g., 08:00, the average ICC included T1‐T2, T1‐T3, T1‐T4, T1‐T5, and T1‐T6). This allowed for a systematic assessment of how reliably each time point correlated with others throughout the day.

In parallel, to examine how the inter‐scan interval influenced test–retest reliability, all scan pairs were grouped by the time elapsed between sessions, ranging from 2 to 12 h. This analysis was grounded in the two‐process model of sleep–wake regulation, which suggests that both homeostatic sleep pressure and the circadian process modulate brain activity (Borbély 1982, 2022). By mapping ICC values to specific interval durations, we assessed whether increased temporal separation led to systematic declines in measurement stability.

## 3 Results

### 3.1 Test–Retest Reliability of rs‐fMRI Metrics Across the Day

**FIGURE 1:**
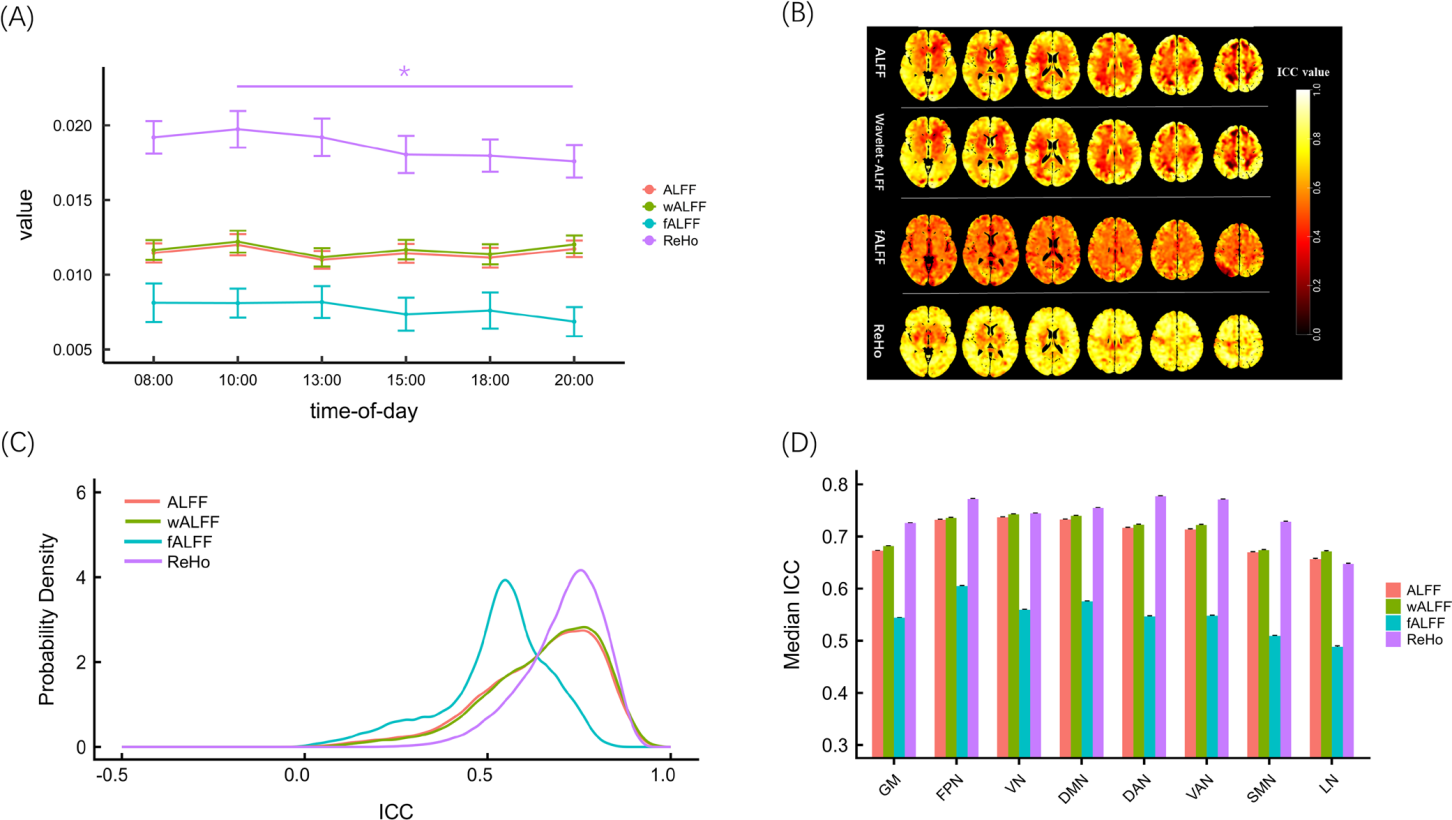
Values and test–retest reliability of rs‐fMRI metrics across time points. (A) Mean values of rs‐fMRI metrics at each of the six time point. (B) ICC maps of rs‐fMRI metrics across six time points. (C) ICC distributions of rs‐fMRI metrics across the six time points; x‐axis indicates ICC values, y‐axis indicates probability density (area under curve = 1). (D) Median ICC values for GM and brain networks of rs‐fMRI metrics. Error bars denote standard errors of the mean. *p < 0.05.

We first explored diurnal variations in rs‐fMRI metrics across the whole‐brain GM. We fitted linear mixed‐effects models (random intercepts for subjects) for each rs‐fMRI metric, with time‐of‐day as the fixed factor and sex and age as covariates. Post hoc pairwise comparisons for time‐of‐day were performed using Tukey tests. The mixed‐effects model revealed a significant main effect of time on ReHo (*F*(5, 190) = 3.65, *p* < 0.01, *η* ^*2*^_*p*_ = 0.05). Post hoc Tukey tests showed that ReHo values at 20:00 were significantly lower than those at 10:00 (*p* < 0.05, Cohen's *d* = 0.76). No significant time‐of‐day effects were observed for ALFF, wALFF, or fALFF (all *p* > 0.05). Group‐level values of rs‐fMRI metrics are shown in Figure 1A.

We next examined the test–retest reliability of these metrics across the six time points using Voxel‐wise ICC. ALFF, wALFF, and ReHo exhibited generally good‐to‐excellent reliability (ICC > 0.6), with ReHo showing the highest reliability overall (see Figure 1B). Voxel‐wise reliability was further assessed using ICC probability density distributions, following the approach of previous work (Yang et al. 2019). As shown in Figure 1C, ReHo showed a rightward‐shifted distribution, indicating greater reliability. To avoid direct quantitative comparisons between metrics with distinct physical meanings, we evaluated the amplitude‐based metrics separately from ReHo. A Friedman test identified significant differences in ICC distributions among the three amplitude‐based metrics (*p* < 0.001). Follow‐up Wilcoxon signed‐rank tests revealed that ALFF and wALFF had significantly higher ICCs compared to fALFF (all *p* < 0.001, Bonferroni corrected).

To further explore spatial variation in reliability, median ICC values were computed with canonical functional networks. As shown in Figure 1D, ReHo consistently yielded robust ICC values across all networks, while fALFF exhibited the lowest among the amplitude‐based metrics. ALFF and wALFF also showed good reliability (median ICC > 0.6) across all networks. Additionally, the LN exhibited significantly lower reliability compared to other networks (all *p* < 0.001, Bonferroni corrected).

### 3.2 Average Test–Retest Reliability at Time Point and Across Scan Intervals

**FIGURE 2:**
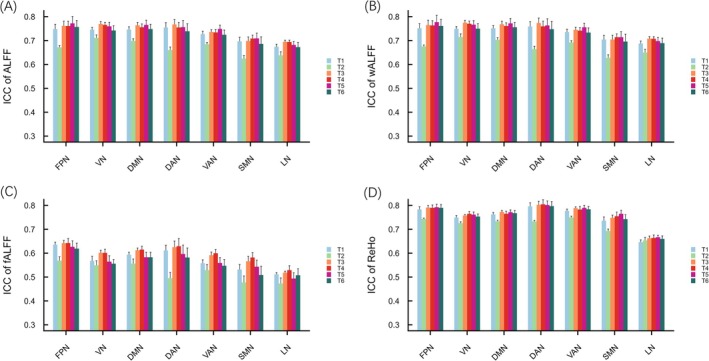
Average test–retest reliability of brain networks across time point. Bar plots depict average ICC values for each network at a given time point, computed relative to the other five time points. Median ICC was used as the reliability index for each network, given its robustness in varying smoothing and cluster size (Caceres et al. 2009). (A) ALFF; (B) wALFF; (C) fALFF; (D) ReHo. Error bars indicate standard errors of the mean.

To evaluate the temporal stability of rs‐fMRI metrics, we first analyzed the average pairwise ICC values for each scanning time point. Across large‐scale networks, scans acquired at 10:00 consistently showed the lowest reliability among all six time points for most metrics (voxel‐wise comparisons, all *p* < 0.001), with the exception of ReHo in the LN (see Figure 2). ReHo exhibited the highest average reliability, with median ICCs exceeding 0.75 across all networks except the LN (Figure 2D).

**FIGURE 3:**
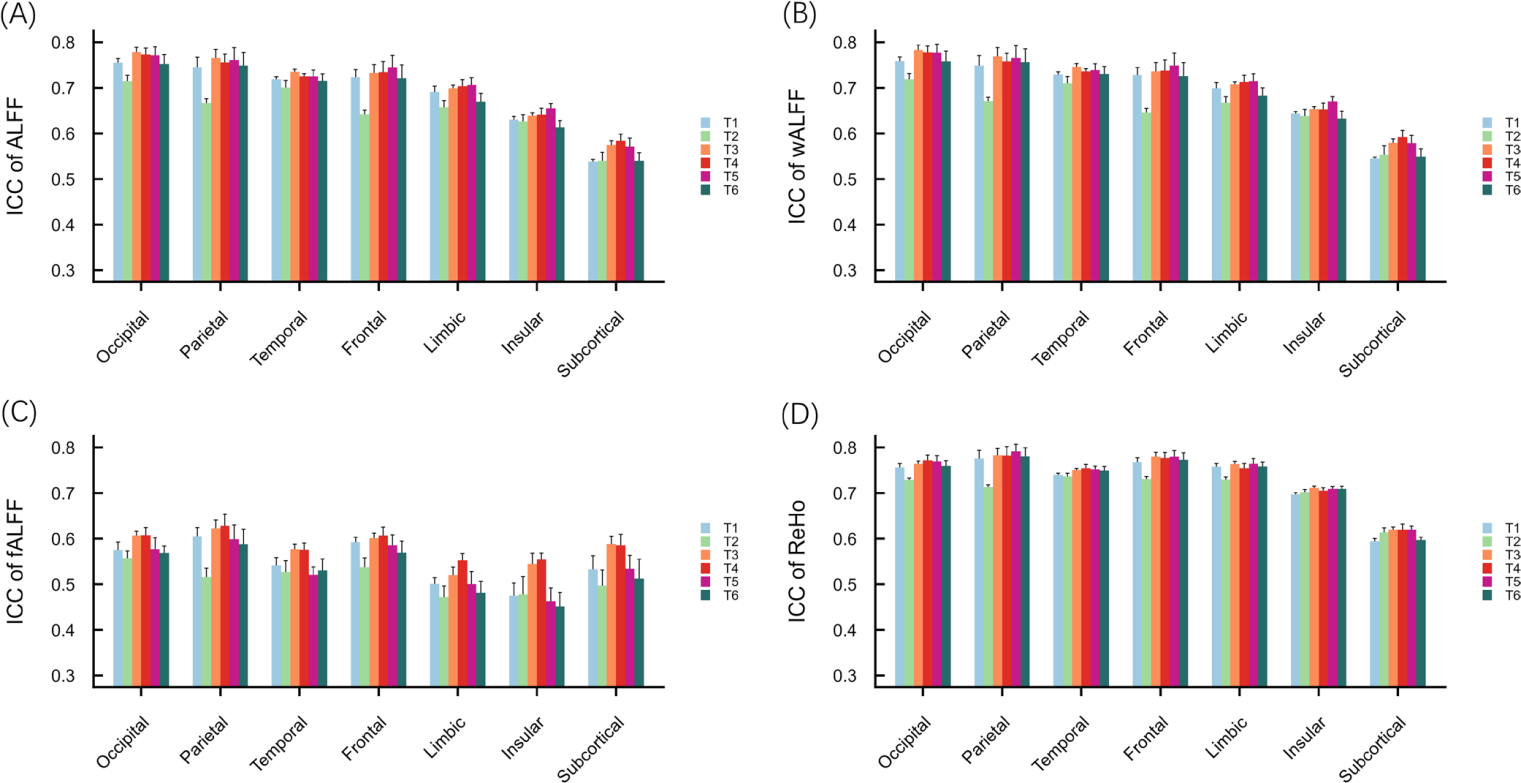
Average test–retest reliability of brain region across time points. Bar plots represent the mean ICC values for each brain region at a given time point, relative to the other five time points. Median ICC was used as the reliability index for each region, given its robustness varying smoothing and cluster size (Caceres et al. 2009). (A) ALFF; (B) wALFF; (C) fALFF; (D) ReHo. Brain regions were defined using the Brainnetome atlas (Fan et al. 2016). Error bars represent standard errors of the mean.

Regional analyses based on the Brainnetome Atlas revealed similar temporal patterns. ALFF, wALFF, and fALFF showed the lowest average reliability at 10:00 in most regions (see Figure 3). In addition, the subcortical nuclei showed the lowest reliability values for ALFF, wALFF, and ReHo among the seven examined networks (*p* < 0.001).

**FIGURE 4:**
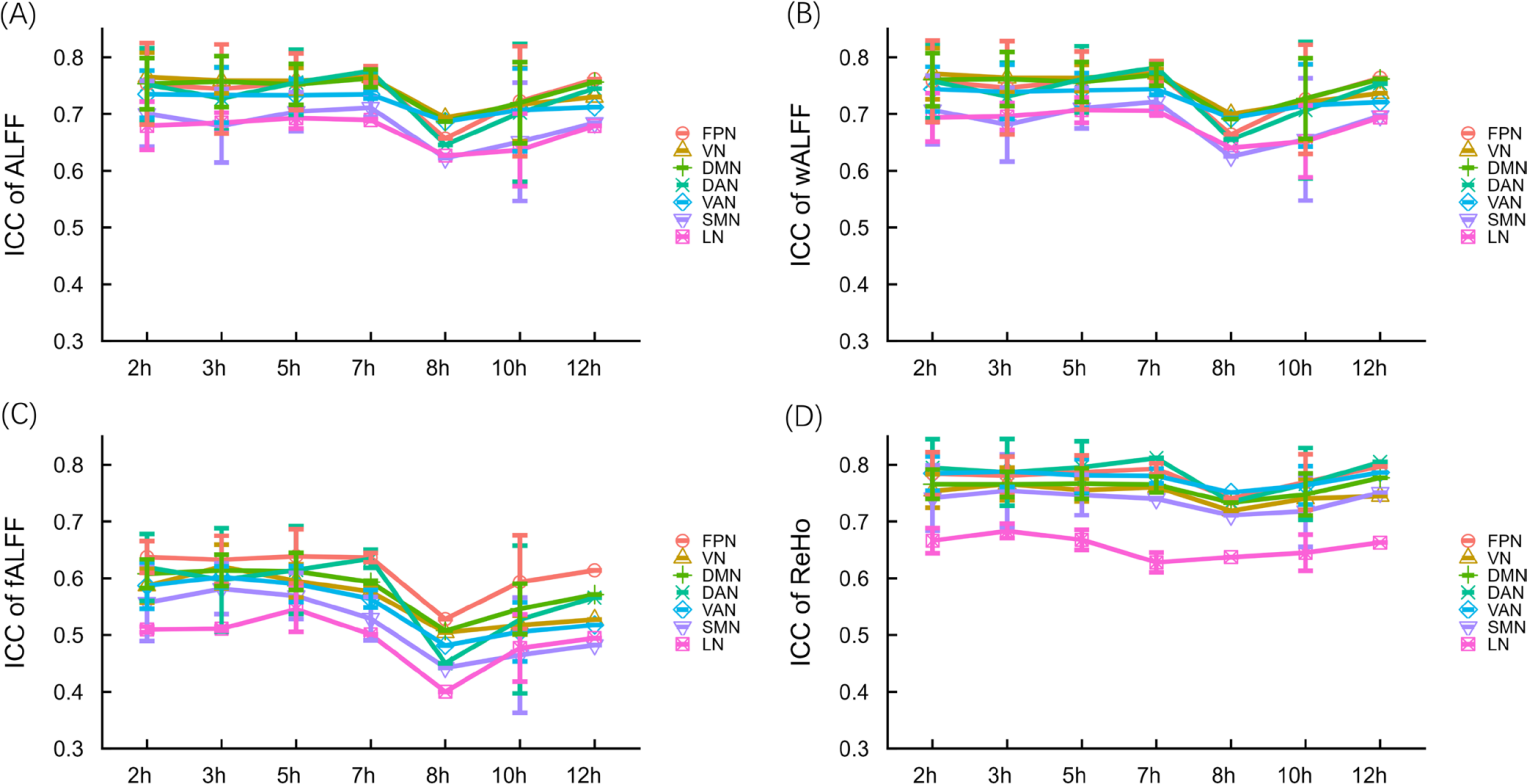
Test–retest reliability of rs‐fMRI metrics across varying inter‐scan intervals in brain networks. ICC values were calculated between each pair of scans and grouped according to the time interval between them. (A) ALFF; (B) wALFF; (C) fALFF; (D) ReHo. Error bars represent standard errors of the mean.

We next explored how the reliability of rs‐fMRI metrics varied with different inter‐scan intervals. When grouped by scan intervals, fALFF showed a general decline in ICC values with increasing interval length, at both global and network levels. However, ALFF, wALFF, and ReHo demonstrated relatively stable ICC values across time intervals (see Figure 4). Voxel‐wise ANOVAs revealed significant main effects of interval time on ICC values for all metrics and networks (all *p* < 0.001). For ALFF, post hoc Tukey–Kramer tests showed significantly lower ICCs at the 12‐h interval compared to the 2‐h interval in GM, VN, SMN, DAN, and VAN. A similar pattern was observed for fALFF across all networks (all *p* < 0.001). For wALFF, ICC values at the 12‐h interval were lower than at the 2‐h interval for GM, VN, SMN, VAN, and FPN (all *p* < 0.01). ReHo exhibited a more heterogeneous pattern: ICCs decreased at the 12‐h interval in GM, VN, VAN, and LN (all *p* < 0.05), but increased in SMN, DAN, FPN, and DMN (all *p* < 0.01).

**FIGURE 5:**
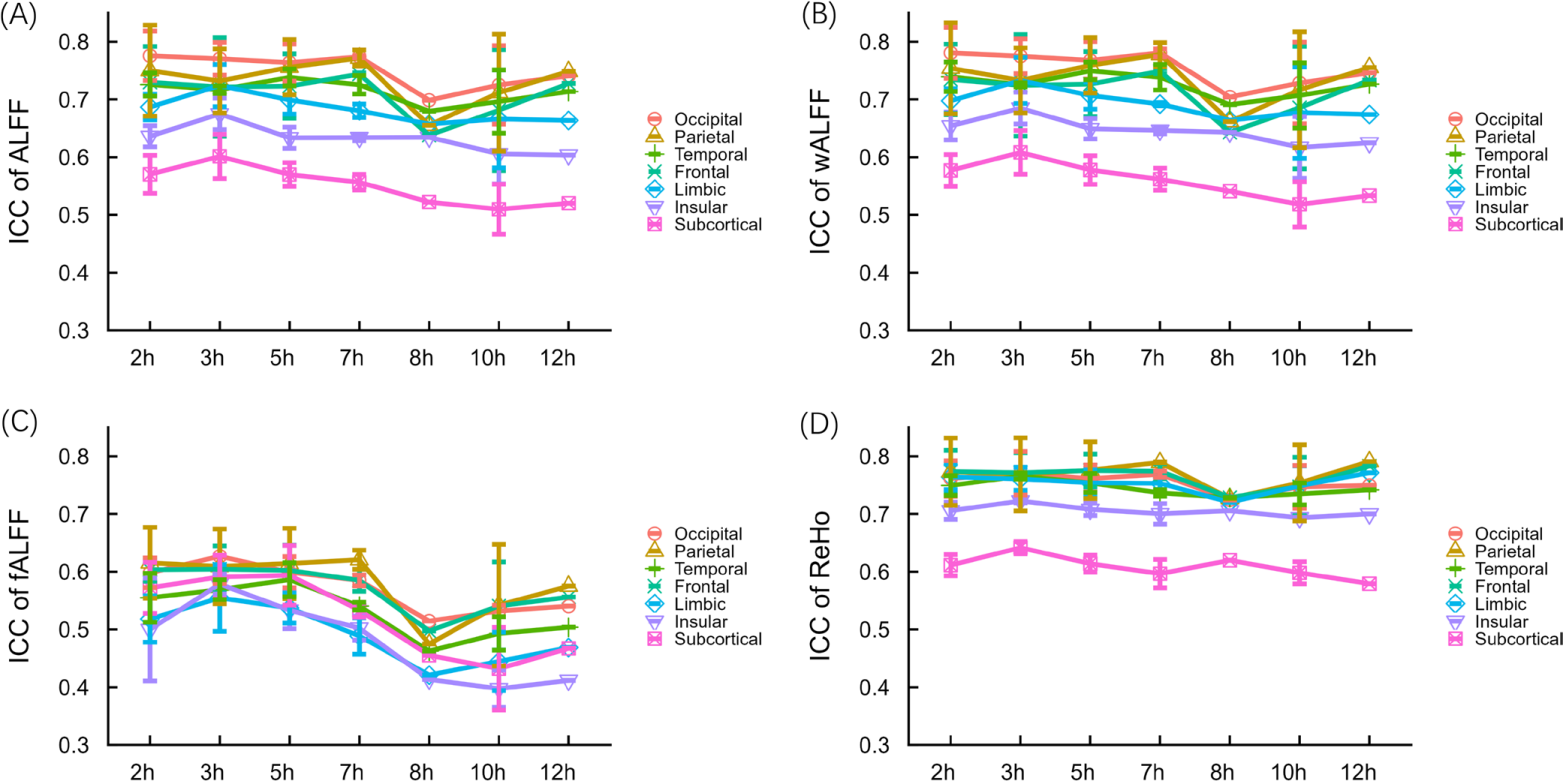
Test–retest reliability of rs‐fMRI metrics across varying inter‐scan intervals in brain regions, based on the Brainnetome atlas (Fan et al. 2016). ICC values were computed between each two scans and grouped according to the inter‐scan interval. (A) ALFF; (B) wALFF; (C) fALFF; (D) ReHo. Error bars represent standard errors of the mean.

At the regional level, similar trends were observed (see Figure 5). ALFF, wALFF, and fALFF showed a monotonic decline in ICCs with increasing scan intervals across most brain regions In contrast, ReHo remained robustly stable across interval time, with minimal fluctuation. Specially, fALFF was particularly sensitive to scan interval, with significant decrease in ICC from 2 to 12 h intervals in nearly all regions (all *p* < 0.001).

### 3.3 Time‐of‐Day Variations in rs‐fMRI Metrics

**FIGURE 6:**
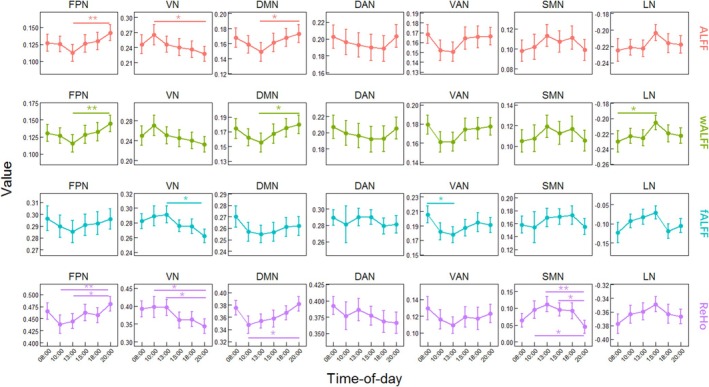
Time‐of‐day variations in rs‐fMRI metrics (Z‐scored). Error bars represent standard errors of the mean. *p < 0.05; **p < 0.01.

We also explored the values of rs‐fMRI metrics across time‐of‐day (see Figure 6). We fitted linear mixed‐effects models with each rs‐fMRI metric as the dependent variable. Time‐of‐day was included as a fixed effect, age and sex were entered as covariates, and subject‐specific random intercepts were included to account for repeated measurements. For ALFF, significant main effects were observed in the FPN and VAN (both *p* < 0.05), with a marginal effect in the DMN (*p* = 0.052). Post hoc Tukey–Kramer tests showed that values at 20:00 were significantly higher than those at 13:00 in both the DMN and FPN (*p* < 0.05). wALFF showed significant time‐of‐day effects in the DMN, FPN, and VAN (all *p* < 0.05), with values at 20:00 exceeding those at 13:00 in both the DMN and FPN (both *p* < 0.05). For fALFF, significant main effects were found in the VAN, VN, and LN (all *p* < 0.05). Post hoc tests showed significant differences between 08:00 and 13:00 in the VAN, and between 13:00 and 20:00 in the VN (both *p* < 0.05). ReHo values also varied across time of day, with significant main effects observed in the DMN, FPN, SMN, and VN (all *p* < 0.05). Post hoc tests revealed that ReHo at 20:00 was higher than that at 10:00 in both the DMN and FPN, while the VN and SMN showed higher values at 10:00 and 13:00 relative to 20:00 (all *p* < 0.05).

## 4 Discussion

In this study, we collected rs‐fMRI data at six distinct time points throughout the day to assess the within‐day reliability of various rs‐fMRI metrics and to investigate the influence of time‐of‐day on their reliability. Among the metrics evaluated, ReHo exhibited a relatively stable ICC profile across sessions, whereas ALFF‐derived measures showed greater temporal variability, with fALFF displaying the largest fluctuations among the amplitude‐based measures. However, ReHo and ALFF‐derived measures should not be regarded as physiologically equivalent, as they reflect distinct aspects of spontaneous BOLD activity. Given these methodological differences, the relative stability of ReHo may partly reflect its rank‐based estimation of local temporal concordance, which may be less sensitive to certain physiological artifacts and extreme signal fluctuations (Zuo et al. 2013; Zuo and Xing 2014). Consistent with this interpretation, Zuo and Xing (2014) reported that ReHo showed relatively high reliability among eight voxel‐wise rs‐fMRI metrics, ranking second only to Dual Regression Independent Component Analysis. In contrast, fALFF exhibited the lowest reliability. Previous research has shown that physiological noise can significantly impact the estimation of amplitude‐based rs‐fMRI measures (Zuo, Di Martino, Kelly, et al. 2010). Since fALFF is more vulnerable to noise interference (Zuo et al. 2014), it is reasonable that fALFF demonstrated the lowest reliability. Despite ReHo's excellent reliability, the present study revealed a diurnal decline in global ReHo, with significantly higher ReHo observed at 10:00 compared to 20:00. This finding aligns with prior research suggesting that intrinsic brain activity is modulated by circadian rhythms (Hodkinson et al. 2014; Kaufmann et al. 2016). Kaufmann et al. (2016) reported stronger default mode network (DMN) connectivity in the morning, which could account for the elevated morning ReHo observed here, as ReHo reflects local synchronization. This enhancement may be associated with circadian‐driven cortical excitability peaks following sleep‐related synaptic renormalization (Vyazovskiy et al. 2011).

Regarding network‐level reliability, the LN demonstrated the lowest test–retest consistency. The LN is closely linked to circadian rhythms (Cardinali 2017; Mivalt et al. 2023). For example, the hippocampus, a key structure of the limbic system, interacts with the suprachiasmatic nucleus (SCN), the master biological clock (Federico et al. 2022), which may introduce greater variability across scans. Zuo and Xing (2014) observed that heteromodal associative networks (default, control, attention) were the most reliable across days, while the limbic network showed the lowest reliability, possibly due to poor rs‐fMRI signal quality or signal loss. Similarly, in the present study, the limbic network exhibited the lowest reliability within a single day, likely due to both low signal quality and circadian modulation. Regarding brain region‐level reliability, the subcortical nuclei, including the thalamus, exhibited the lowest reliability across the Brainnetome Atlas. The thalamus is known to interact closely with the SCN and plays a crucial role in arousal and circadian regulation (Avanzini et al. 2000; Colavito et al. 2015; Xing et al. 2023), which may explain the pronounced diurnal variability and reduced test–retest reliability observed in this region.

Across most brain networks and regions, the lowest test–retest reliability was observed at 10:00 for all metrics, suggesting greater signal variability at this time point compared to the others. Two possible factors may explain this finding. First, it may be related to the circadian neuroendocrine transition, particularly cortisol decline. The 10:00 time point coincides with the post‐peak decline phase of cortisol, which peaks 30–45 min after awakening. Rapid cortisol decline disrupts neurovascular coupling in prefrontal‐limbic circuits, increasing BOLD signal variability (Duan et al. 2025; Jiang et al. 2017). Second, the variability may reflect fluctuations in attentional state and arousal. Morning hours typically involve rapid shifts from sleep inertia to sustained alertness. By 10:00, some participants may still experience residual sleep inertia, while others achieve full alertness. This variability in individual arousal states could contribute to greater signal variability, particularly in arousal‐sensitive regions like the anterior cingulate, a key node of the salience network (Liu et al. 2018; Yang et al. 2023; Zhang et al. 2024).

Regarding reliability across different time intervals, ICC values for most regions and networks showed a decreasing trend for ALFF, wALFF, and fALFF, indicating that longer inter‐scan intervals reduce measurement stability. This decline is consistent with the influence of homeostatic sleep pressure (Process S), as extended intervals allow greater shifts in brain activity due to accumulating sleep pressure. These results align with prior evidence from resting‐state and task‐based CBF studies showing time‐dependent reductions in reliability (Guo et al. 2024). In contrast, ReHo remained relatively stable across all intervals, underscoring its robustness for within‐day experimental designs.

We also observed distinct diurnal patterns across networks. rs‐fMRI metrics in the frontoparietal and default mode networks were elevated at 20:00 compared with 13:00 or 10:00, whereas the visual network exhibited the opposite trend. This pattern is consistent with prior evidence of reduced BOLD signal variance in the visual cortex at twilight compared to midday, interpreted as adaptive quieting in preparation for diminished sensory input (Cordani et al. 2018). In contrast, the evening increases in ALFF and ReHo within the FPN and DMN likely reflect heightened intrinsic cognitive processing. In line with this interpretation, Xing et al. (2023) reported elevated fALFF and ReHo in the core nodes of the FPN and DMN during evening hours relative to midday.

Several limitations of this study should be noted. First, physiological variables were not recorded, including heart rate, blood pressure, body temperature, hormonal levels (cortisol, melatonin), as well as menstrual cycle phase and contraceptive use in female participants. Incorporating these variables in future studies is crucial to identify the physiological drivers of diurnal brain variations and account for their potential influence. In particular, future studies focusing on the limbic system or other hormone‐sensitive regions should strictly control for menstrual cycle phase and hormonal contraceptive status, given evidence that ovarian hormones modulate cortico‐limbic and subcortical brain measures (Avila‐Varela et al. 2024; Dubol et al. 2021). Second, while our within‐day fixed‐order schedule follows common diurnal/circadian neuroimaging paradigms (e.g., Carlucci et al. 2023; Muto et al. 2016; Park et al. 2012; Xing et al. 2023) and reduces inter‐day variability, it cannot fully disentangle diurnal effects from residual order‐related influences and their possible interactions with age and sex. Moreover, participants may become more familiar with the scanning environment across sessions, potentially reflecting a practice or habituation effect. Such habituation may partly offset fatigue‐related physiological changes. Although existing evidence suggests these influences (e.g., fatigue, expectancy, habituation) are generally modest in resting‐state paradigms (Bermpohl et al. 2006; Chapman et al. 2010; Chen et al. 2015; Honey et al. 2009; McGlynn et al. 2007; Nakagawa et al. 2013; Qi et al. 2019; Schumann et al. 2021; Tsai et al. 2024), direct evidence on their influence on brain diurnal variations remains limited. Future studies with counterbalanced orders are warranted. Third, although participants were instructed to maintain regular sleep–wake schedules, the use of polysomnography (PSG) in a sleep laboratory would have provided a more precise evaluation of sleep history and enabled direct examination of links between sleep EEG and fMRI variability across the day. Finally, this study focused exclusively on healthy young adults, which may limit the generalizability of the findings to other populations. Expanding future research to include broader age ranges and diverse clinical groups will improve the external validity of these results.

## 5 Conclusion

In summary, this study provides the first comprehensive evaluation of within‐day test–retest reliability for four widely used resting‐state fMRI metrics. ReHo exhibited a relatively stable reliability profile across sessions, whereas amplitude‐based measures showed greater temporal variability, with fALFF displaying the largest fluctuations. Reliability across most brain regions and networks showed a pronounced decline at 10:00, suggesting a mid‐morning circadian disruption. Moreover, reliability declined with longer inter‐scan intervals for amplitude‐based measures, but not for ReHo, which remained stable throughout the day. These findings underscore the critical influence of time‐of‐day on the reproducibility of resting‐state fMRI measures and highlight the need to account for circadian factors in both experimental design and data interpretation.

## Funding

This research was supported in part by the grants from the Brain Science and Brain‐like Intelligence Technology—National Science and Technology Major Project (2021ZD0200500), the National Natural Science Foundation of China (32441108, 32200889), the Shanghai International Studies University Research Projects (2021114002), the Open Research Fund of the State Key Laboratory of Cognitive Science and Mental Health, the Zhejiang Provincial Natural Science Foundation of China (LQN25G020003), and the Major Humanities and Social Research Projects in Zhejiang higher education institutions (2024QN071).

## Conflicts of Interest

The authors declare no conflicts of interest.

## Data Availability

The data and code used in the present study are available from the corresponding author upon request.
